# Efficacy of Cranial Orthosis for Plagiocephaly Based on 2D and 3D Evaluation

**DOI:** 10.1055/a-2222-1494

**Published:** 2024-01-24

**Authors:** Hiroki Kajita, Ichiro Tanaka, Hiroaki Komuro, Shigeru Nishimaki, Isao Kusakawa, Koichiro Sakamoto

**Affiliations:** 1 Baby's Head Reshaping Clinic in Tokyo, Japan; 2 Department of Plastic and Reconstructive Surgery, Keio University School of Medicine, Tokyo, Japan; 3 Department of Plastic and Reconstructive Surgery, Tokyo Dental College Ichikawa General Hospital, Tokyo, Japan; 4 Department of Pediatrics, Yokohama City University Hospital, Yokohama, Japan

**Keywords:** imaging, three-dimensional, orthotic devices, photogrammetry, plagiocephaly, nonsynostotic

## Abstract

**Background** With the advent of cranial orthoses as therapeutic medical devices for the treatment of severe positional head deformities in Japan, an increasing number of patients are being treated with them. However, assessing the effectiveness of a treatment is often difficult due to the use of different metrics. This study aimed to evaluate the effectiveness of cranial orthoses for deformational plagiocephaly using two- (2D) and three-dimensional (3D) evaluation metrics.

**Methods** We conducted a retrospective study of infant patients with deformational plagiocephaly who underwent cranial orthosis treatment. We evaluated the severity of deformational plagiocephaly using cranial asymmetry (CA) and the cranial vault asymmetry index (CVAI) as 2D metrics, and anterior and posterior symmetry ratios as 3D metrics. The patients were divided into 24 subgroups based on the initial severity of each outcome and their age at the start of treatment. We analyzed the changes in outcomes and correlations within improvements across the age and severity categories.

**Results** Overall, 1,038 infants were included in this study. The mean CA, CVAI, and anterior and posterior symmetry ratios improved significantly after cranial orthosis treatment. The improvement in each score was greater in patients with more severe initial deformities and in those who underwent treatment at a younger age.

**Conclusion** Cranial orthosis treatment was effective in correcting deformational plagiocephaly in infants, as demonstrated by improvements in both 2D and 3D metrics. Patients with more severe initial deformities and those who underwent treatment at a younger age showed greater improvement.

## Introduction

Deformational plagiocephaly is a condition in which the skull shape gets deformed during the fetal period and infancy due to mechanical factors. 1 This deformation is caused by mechanical factors that affect skull growth, such as the baby turning over or spending time in the same position. Although deformational plagiocephaly is generally considered medically benign and naturally gets better to some extent, some cases may require treatment to correct the shape.

Deformational plagiocephaly is common in Japan due to the cultural practices that involve placing infants on their backs to sleep and carrying them on their backs. 2 These practices can exert pressure on the back of the head and result in its flattening. Historically, this was considered a sign of a well-behaved baby, and little concern was given about its potential long-term effects on the child's development. However, recent research has shown the importance of early intervention in correcting deformational plagiocephaly. 3

According to the Guidelines for the Management of Patients with Positional Plagiocephaly published by the Congress of Neurological Surgeons and the Section on Pediatric Neurosurgery, 3 repositioning therapy is typically recommended as the first-line treatment option for deformational plagiocephaly. Helmet therapy may be considered for infants with persistent moderate-to-severe plagiocephaly after a course of conservative treatment, and it is also recommended for infants with moderate-to-severe plagiocephaly presenting at an advanced age.

In Japan, helmet therapy for positional head deformities was introduced in 2007 by Aihara et al, 4 and some cranial orthoses have been approved as medical devices by the Pharmaceuticals and Medical Devices Agency since 2018. Recently, a few Japanese studies have provided evidence regarding the effectiveness of helmet therapy. 2 5

In general, infants with more severe initial deformities and those who receive helmet therapy earlier in infancy tend to have a greater chance of achieving better correction and normalization of head shape. 4 6 7 However, the studies used different devices and outcome metrics (such as two-dimensional [2D] and three-dimensional [3D] parameters), making it difficult to compare their results with those of other studies. 2 Nonetheless, relying solely on 2D evaluation may not fully assess the overall 3D structure of the skull, and 3D evaluation provides a detailed assessment of cranial shape from multiple perspectives. 8 9 10 Therefore, it is necessary to demonstrate the extent to which 2D metrics, which have become prevalent, and 3D metrics improve for each month of age and severity.

This study aimed to examine the impact of age and severity of deformity on the effectiveness of cranial orthosis treatment in infant patients with deformational plagiocephaly, as measured using both 2D and 3D metrics. This study also aimed to demonstrate that younger and more severe cases exhibit greater improvements in 3D evaluation. Providing details on the effectiveness of treatment for different age groups and severity levels can help medical professionals and the families of patients anticipate realistic treatment outcomes.

## Methods

### Study Design and Patients

**Fig. 1:**
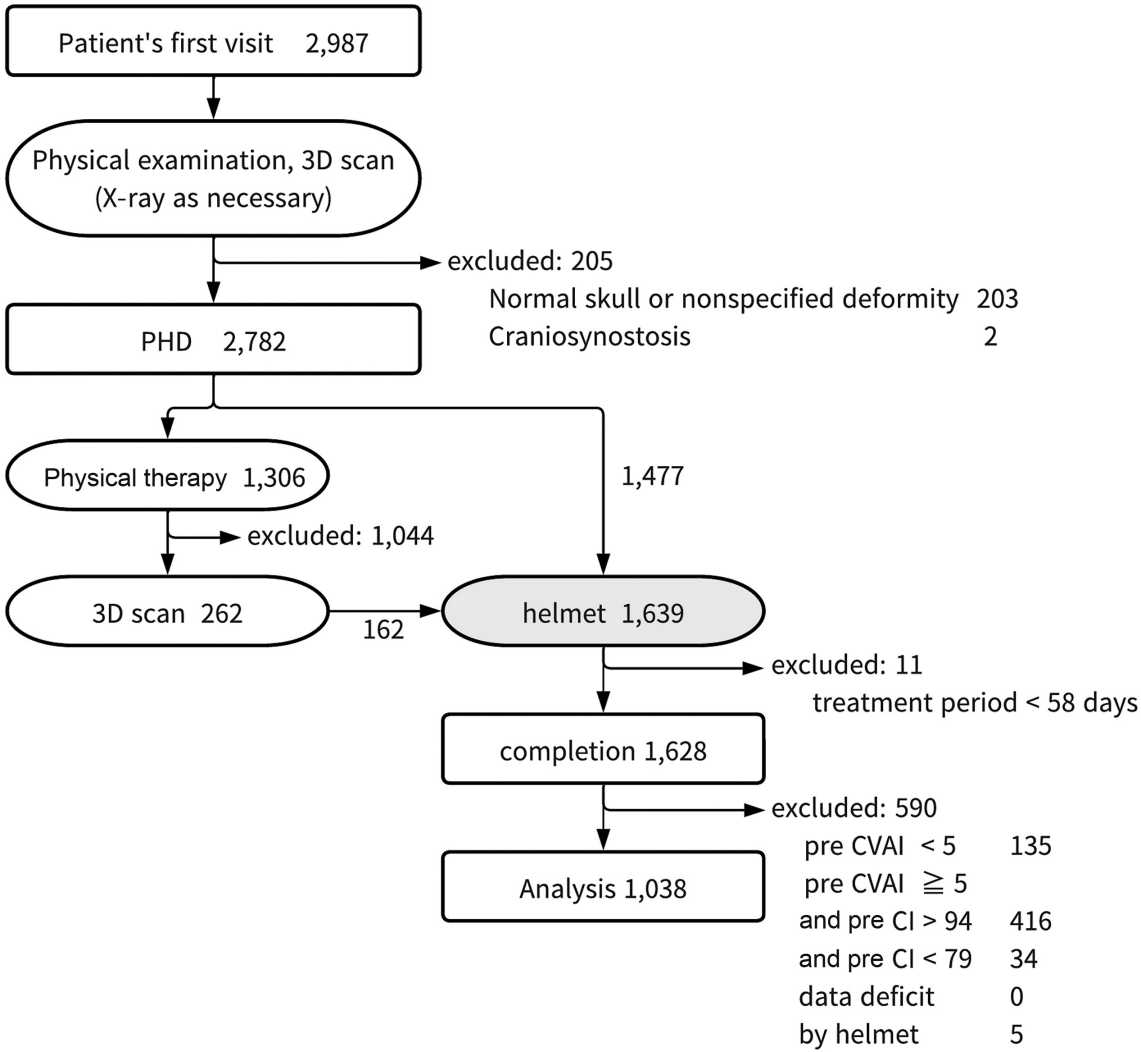
Treatment algorithm. CI, cephalic index; CVAI, cranial vault asymmetry index; PHD, positional head deformity; 3D, three-dimensional.

This was a single-arm, retrospective, nonrandomized study without a control group of untreated infants. The study included infant patients who visited our clinic for cranial deformities between March 4, 2021, and October 31, 2022. The clinic is staffed by a team of highly qualified board-certified medical professionals, including two plastic surgeons, three neurosurgeons, two pediatric surgeons, and three pediatricians. The patients were evaluated and treated according to the algorithm shown in Fig. 1 .

The inclusion criteria for this study were the presence of nonsynostotic plagiocephaly, regular follow-up during helmet therapy, with complete documentation. This study enrolled infants with isolated plagiocephaly, with cranial vault asymmetry index (CVAI) > 5.0. 11 The exclusion criteria for the study were the presence of brachycephaly (cephalic index [CI] > 94) or scaphocephaly (CI < 79), 12 a treatment duration of <58 days, or the use of a different helmet device. As the shortest duration for completing helmet therapy among the included patients was 58 days, all patients who had longer periods of treatment were included in this study. The patients were divided into subgroups based on the severity of their presentation and age at the start of treatment. The study was conducted in accordance with the principles of the Declaration of Helsinki. The protocol was approved by the Institutional Review Board (approval number: T-22001) with a waiver of consent.

### Data Acquisition Using a Three-Dimensional Scanner

**Fig. 2:**
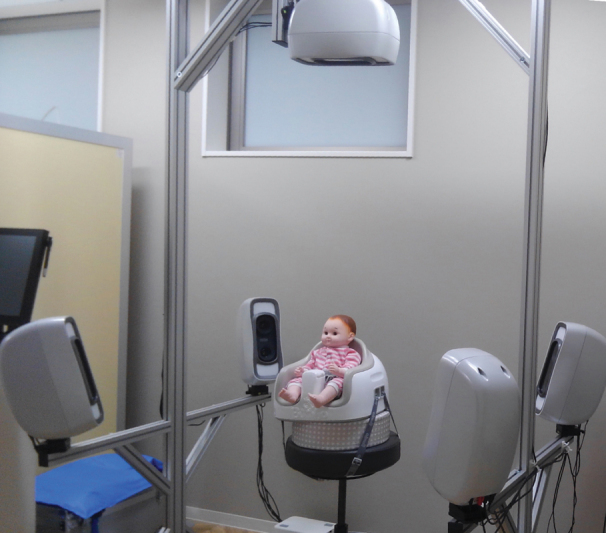
VECTRA-M5 360° 3D scanner.

We conducted a comprehensive 360-degree scan of the cranial shape, including both ears, using the VECTRA-M5 360° 3D scanner (Canfield Scientific Inc., Parsippany, NJ). 13 This scanner uses five synchronized cameras to perform stereophotogrammetric imaging ( Fig. 2 ), allowing for contact-free data acquisition in <2 milliseconds. This minimizes movement artifacts, making it ideal for data acquisition, especially for infants. While validation tests of the scanner have been conducted on a mannequin head, 14 healthy adults, 15 patients with cleft lips or palates, 16 17 and infant's head, 18 it has not yet received approval for use as a medical device by the Pharmaceuticals and Medical Devices Agency in Japan. The VECTRA-M5 360° 3D scanner was calibrated daily following the manufacturer's instructions to ensure the accurate geometric configuration of all cameras. This process is essential for establishing and recording the relationship between the cameras and other components in the system. An elastic wig cap was used to cover the infants' hair and prevent the failure of data acquisition.

### Data Analysis Method

**Fig. 3:**
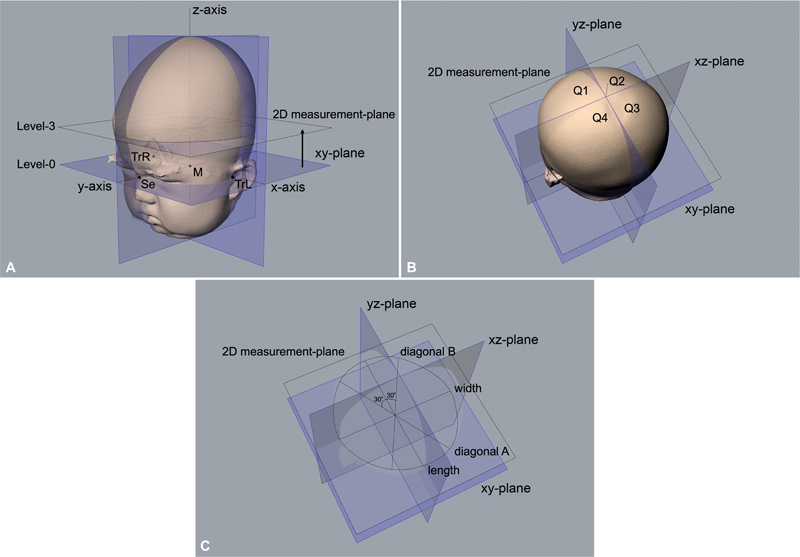
Three-dimensional images of an infant's head. ( **A** ) How the coordinate system is defined. ( **B** ) The cranial vault is separated into four volumes by the planes of the coordinate system. ( **C** ) Two-dimensional measurement plane is set at the height of three-tenths from the xy-plane to the top of the head. M, midpoint between the tragions; Q, quadrant; Se, sellion; TrL, left tragion; TrR, right tragion; 2D, two-dimensional.

A specialized image analysis software (Japan Medical Company Inc., Tokyo, Japan) was used to align and analyze the obtained 3D images ( Fig. 3 ). To define the coordinate system in virtual space, three anatomically defined reference points were used: the left tragion, the right tragion, and the sellion. The alignment procedure was performed as described in previous studies. 4 5 8 9 10 19 20 Subsequently, we identified the basic cross-section (xy plane) passing through the sellion and the left and right tragions. The origin was defined as the midpoint between the two tragions. After setting these landmarks and establishing the basic plane, we defined the y-axis as the line passing through the sellion and origin, and the x-axis was defined as the line perpendicular to the y-axis, intersecting the origin on the basic plane. The z-axis was defined as the line perpendicular to the xy plane, intersecting the origin. 4 5 10 21 We constructed 10 evenly spaced parallel cross-sections through the upper portion of the head (level 10) from the xy plane (level 0). The volume of the entire head, excluding the ear and face regions, was calculated using cross-sections from levels 2 to 8. 22 The 2D and 3D parameters were calculated subsequently.

#### Two-Dimensional Measurement

In this study, level 3 was chosen as the standard measurement plane for the subsequent 2D evaluations ( Fig. 3C ). A decision was made to standardize the levels of the measurement plane. Cranial asymmetry (CA) in millimeters (mm) was calculated as the difference between the two diagonal cranial diameters, measured at an angle of 30 degrees from the y-axis. 11 CA was calculated as diagonals A minus B. CVAI (%) was calculated as CA divided by diagonal B and then multiplied by 100 (where diagonal A > B). To calculate CI (%), the width of the head was divided by its length and then multiplied by 100. The resulting CI was used to exclude patients who were brachycephalic (>94%) or scaphocephalic (CI < 79%).

#### Three-Dimensional Measurement

The total volume of the head was separated into four quadrants using planes that passed through the x- and y-axes and contained the z-axis (xz and yz planes) ( Fig. 3B ). The quadrants were labeled as follows: Q1 (anterior left), Q2 (anterior right), Q3 (posterior right), and Q4 (posterior left). The volume of each quadrant was then used to calculate the bilateral symmetry ratio, which consisted of the anterior symmetry ratio (ASR) and the posterior symmetry ratio (PSR). 5 10 ASR (%) was calculated as the volume of Q1 divided by that of Q2 (or vice versa) and then multiplied by 100; PSR (%) was calculated as the volume of Q3 divided by that of Q4 (or vice versa), then multiplied by 100. For the calculation, a value of <100% was chosen for either the Q1/Q2 or Q3/Q4 ratios (or vice versa). According to the aforementioned method, the numerical values of CI, ASR, and PSR have been reported to be more accurate than those of CA. 10

#### Severity Classifications

The severity classification of CVAI was defined as follows: mild (5 to <7%), moderate (7 to <9%), severe (10 to <13%), and very severe (≥14%). 2 The severity classification of CA was as follows: mild (6 to <9 mm), moderate (9 to <12 mm), severe (13 to <16 mm), and very severe (≥17 mm). The severity classifications of ASR and PSR were defined as follows: level 1 (≥90%), level 2 (85–90%), level 3 (80–85%), and level 4 (≤80%).

### Helmet Therapy

After excluding craniosynostosis through a physical examination, with or without X-ray confirmation, helmet therapy was considered for infants with persistent plagiocephaly in which the family reported that no improvement was seen with measures taken at home and those with moderate-to-severe plagiocephaly presenting at an advanced age over 6 months. Subsequently, a custom-made cranial orthosis (Aimet®, Japan Medical Company Inc., Tokyo, Japan; medical device approval number: 30100BZX00022000) was introduced upon request from the parents or guardians. After the initial fitting of the helmet, the family was instructed to have the patient wear the helmet for 23 hours a day, with a gradual increase in wearing time during a 7- to 14-day break-in period. They were also advised to visit the clinic for follow-up scans and helmet adjustments after 3 to 4 weeks to accommodate head growth and changes in skull shape. The helmet was to be worn continuously until it became tight or until the parents were satisfied with the shape of the patient's head. If patients experienced any side effects, such as skin injuries from wearing the helmet, parents were advised to bring them to the clinic.

### Statistical Analyses

This was a single-arm, retrospective, nonrandomized study that did not include a control group of untreated patients. The primary outcomes assessed in this study were the improvements in ASR and PSR before and after helmet therapy. Additionally, improvements in CVAI and CA were also assessed. The total duration of helmet therapy was recorded and analyzed in relation to the effectiveness of the treatment and the age of the patients. The null hypothesis was defined as no correlation between age, severity, or improvement in ASR and PSR. The mean treatment duration and standard deviation (SD) were determined for each subgroup based on severity and age at the beginning of treatment. To account for multiple testing of the subgroups, the significance level (α) was adjusted from 0.05 to 0.0083 (for age) or 0.0125 (for severity) using the Bonferroni correction. Kendall's rank correlation coefficient was used to assess trends in parameter improvement across age and severity categories. Statistical analyses were performed using the SciPy software (version 1.10.1; www.scipy.org ). 23

## Results

### Clinical Characteristics and Measured Values

During the 20-month period, a total of 2,987 patients (1,863 boys and 1,124 girls) visited the clinic. Among them, 2 patients were diagnosed with craniosynostosis, and 203 had a normal skull without any positional head deformities (CA < 6 mm, CVAI < 5%, ASR < 90%, PSR < 90%, and 80% < CI < 94%). Among the 2,779 patients with positional head deformities (including 161 who underwent physical therapy), helmet therapy was recommended to 1,639 patients whose parents were willing for it ( Table 1 ). Helmet therapy was initiated 2 weeks after the initial visit. However, after 58 days of treatment, nine patients did not attend follow-up visits. For further analysis, the focus was on the 1,038 patients with isolated plagiocephaly who completed helmet therapy using Aimet ^®^ and did not have brachycephaly ( *n* = 416) or scaphocephaly ( *n* = 34; Fig. 1 ).

**Table 1:** Clinical characteristics of the patients and treatment results ( *n* = 1,038)

Outcomes	Classification	Definition	Before helmet therapy	Percentage (%)	After helmet therapy	Percentage (%)	*p* -Value a
CA	Normal	0–5	0	(0%)	291	(28.0%)	
	Mild	6–8	38	(3.7%)	401	(38.6%)	
	Moderate	9–12	274	(26.4%)	257	(24.8%)	
	Severe	13–16	417	(40.2%)	80	(7.7%)	
	Very severe	≥17	309	(29.8%)	9	(0.9%)	
	Mean: CA (SD)		15.11	(SD 3.8)	7.93	(SD 3.4)	<0.001
CVAI	Normal	0–4	0	(0%)	531	(51.2%)	
	Mild	5–6	86	(8.3%)	326	(31.4%)	
	Moderate	7–9	399	(38.4%)	160	(15.4%)	
	Severe	10–13	475	(45.8%)	21	(2.0%)	
	Very severe	≥14	78	(7.5%)	0	(0%)	
	CVAI, mean (SD)		10.32	(SD 2.4)	5.15	(SD 2.1)	<0.001
ASR	Level 1	>90	610	(58.8%)	956	(92.1%)	
	Level 2	86–90	309	(29.8%)	71	(6.8%)	
	Level 3	81–85	97	(9.3%)	11	(1.1%)	
	Level 4	≤80	22	(2.1%)	0	(0%)	
	ASR, mean (SD)		90.68	(SD 4.7)	95.32	(SD 3.6)	<0.001
PSR	Level 1	>90	38	(3.7%)	543	(52.3%)	
	Level 2	86–90	193	(18.6%)	347	(33.4%)	
	Level 3	81–85	346	(33.3%)	123	(11.8%)	
	Level 4	≤80	461	(44.4%)	25	(2.4%)	
	PSR, mean (SD)		80.63	(SD 5.9)	89.75	(SD 4.6)	<0.001

Abbreviations: ASR, anterior symmetry ratio; CA, cranial asymmetry; CVAI, cranial vault asymmetry index; PSR, posterior symmetry ratio; SD, standard deviation.

The number of patients for each outcome severity level before and after treatment, along with the mean and standard deviation for each outcome, are shown. Values before and after treatment were compared using the paired *t* -test.

a Paired *t* -test.

The mean age of the patients at the time of the scan immediately before starting helmet therapy was 21.5 (SD 7.0) weeks, and the mean treatment duration was 22.4 (SD, 6.0) weeks. Prior to helmet therapy, the mean CA was 15.1 mm, CVAI was 10.3%, ASR was 90.7%, and PSR was 80.6% ( Table 1 ). Following treatment, there was a statistically significant improvement in ASR, PSR, CA, and CVAI ( *p* < 0.001, paired *t* -test).

### Relationship between the Outcomes

**Fig. 4:**
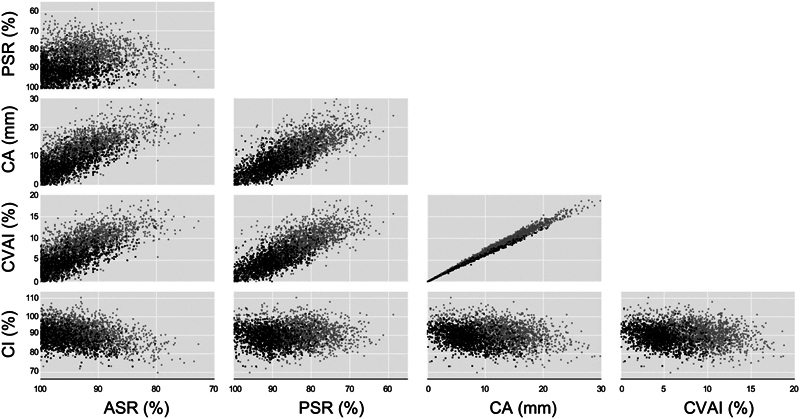
Distribution map of ASR, PSR, CA, and CVAI ( *n* = 1,637). The gray dots represent the data before treatment, and the black dots represent the data after treatment. ASR, anterior symmetry ratio; CA, cranial asymmetry; CI, cephalic index; CVAI, cranial vault asymmetry index; PSR, posterior symmetry ratio.

To visualize the relationship between parameters, distribution maps were created for ASRs, PSRs, CAs, CVAIs, and CIs from 1,637 patients who underwent helmet therapy ( Fig. 4 ). The distribution map revealed that in many patients, PSR was smaller than ASR, while in the remaining patients, ASR was smaller than PSR. It was also observed that some patients exhibited low PSR or ASR despite having normal CVAI and CA values. Conversely, in some patients, PSR and ASR were close to 100%, but CVAI and CA values were relatively higher. Additionally, it was noted that there was a tendency for PSR to be smaller in infants with brachycephaly (larger CI), and for ASR to be smaller in those with scaphocephaly (smaller CI).

### Improvement of the Outcomes

**Fig. 5:**
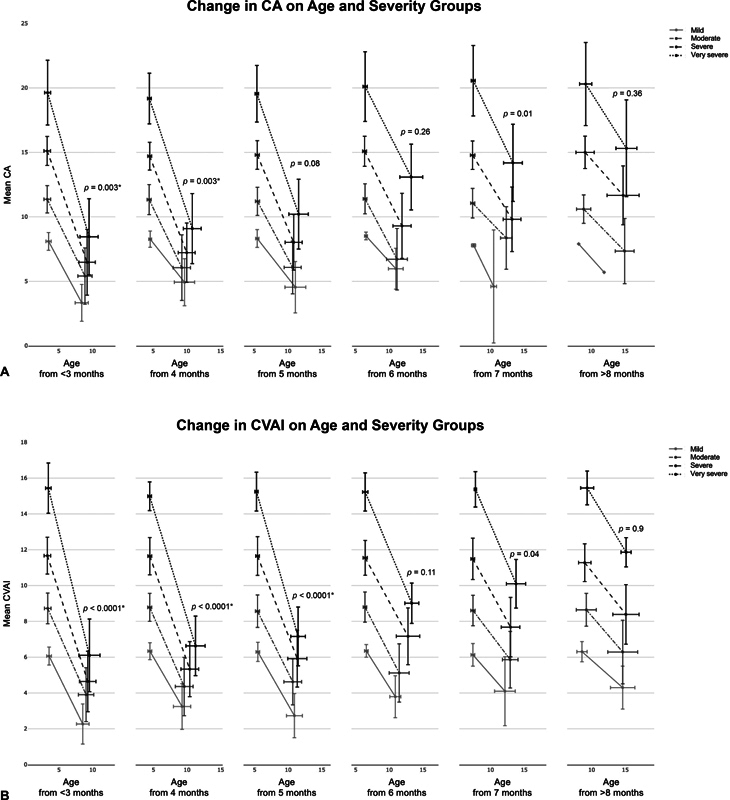
The mean change in outcomes with standard deviations before and after helmet therapy for each subcategory ( *n* = 1,038). The x-axis error bars represent the standard deviation of age, and the y-axis error bars represent the standard deviation of outcomes. The *p* -values are from Kendall's rank correlation test for trend, which shows the trend of the final number within age categories as severity increases. * indicates significance ( *p* < 0.0083). ASR, anterior symmetry ratio; CA, cranial asymmetry; CVAI, cranial vault asymmetry index; PSR, posterior symmetry ratio.

**Fig. 6:**
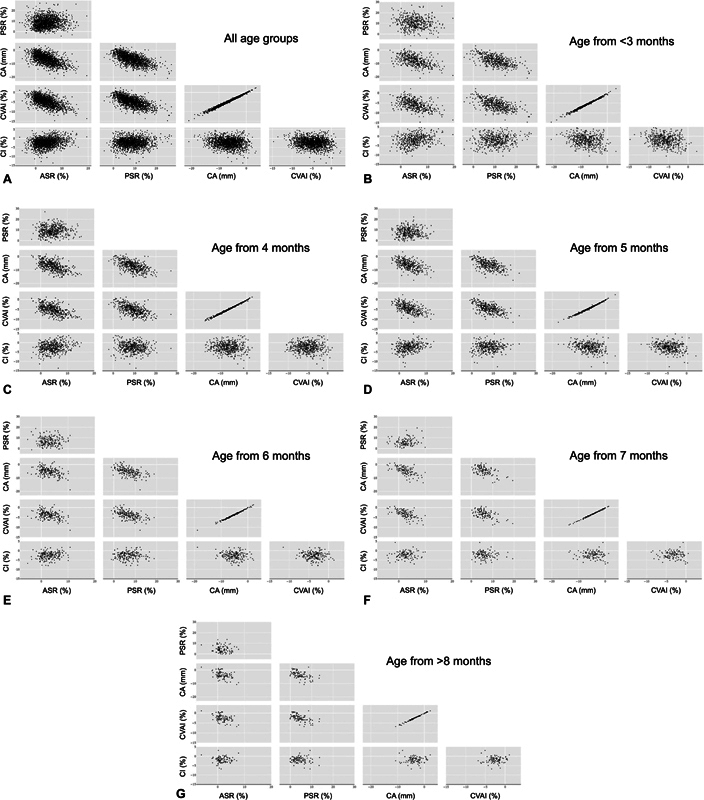
Distribution map of the change in ASR, PSR, CA, and CVAI ( *n* = 1,637) from before to end of helmet therapy. ( **A** ) Scatter matrix for all age groups. ( **B–G** ) Scatter matrices for each age group. ASR, anterior symmetry ratio; CA, cranial asymmetry; CI, cephalic index; CVAI, cranial vault asymmetry index; PSR, posterior symmetry ratio.

The groups were further divided into subgroups based on age and severity, resulting in 24 subcategories for each parameter, including CA, CVAI, ASR, and PSR. The changes in each outcome during treatment were evaluated for each subgroup ( Fig. 5 ). A statistically significant increasing trend was observed in the amount of changes in ASR and PSR with the increasing initial severity across all age categories ( Fig. 5C, D ). This indicates that the more severe the initial condition was in all age groups, the greater the improvement seen in ASR and PSR at the end of the treatment. As for CVAI, similar trends were observed in groups with ages <6 months ( Fig. 5B ). To determine the impact of increasing initial age on the final parameters within each severity category, the same trend test was conducted across the different age groups. The results revealed that an increase in age was generally associated with an increase of CA and CVAI or a decrease in ASR and PSR in the final parameter measurements, except for the PSR level 1 group. The relationship between the change in two of parameters are also visualized for each age group ( Fig. 6 ).

**Figure:**
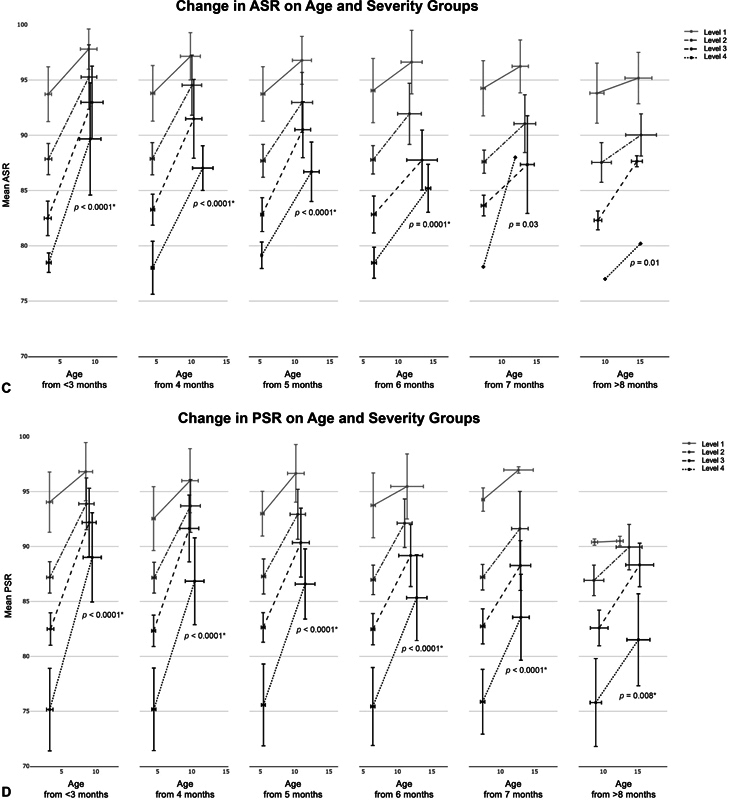


### Treatment Duration

The mean treatment duration and SD were calculated for each subcategory. The division of the participants, their respective mean treatment times, and *p* -values calculated using Kendall's rank correlation test are presented in Table 2 . The treatment duration tended to increase with the severity of PSR in groups with age < 7 months. However, within each isolated severity category, the statistical trend for treatment duration did not exhibit a significant increase with age. This means that starting treatment early does not necessarily shorten the treatment duration.

**Table 2:** Mean treatment durations with their corresponding standard deviations for each age and severity subgroup ( *n* = 1,038)

Outcomes	Initial severity	Age during helmet initiation (months)						p-Trend for severity a
		-3	4	5	6	7	8+	
CA	Mild	4.36 ± 1.06 ( *n* = 15)	4.56 ± 1.4 ( *n* = 10)	5.1 ± 1.54 ( *n* = 7)	3.87 ± 1.17 ( *n* = 4)	3.06 ( *n* = 1)	3.06 ( *n* = 1)	0.71
	Moderate	5.02 ± 1.07 ( *n* = 76)	4.36 ± 1.17 ( *n* = 83)	4.71 ± 1.26 ( *n* = 52)	4.29 ± 1.49 ( *n* = 32)	4.48 ± 0.91 ( *n* = 19)	5.51 ± 1.07 ( *n* = 12)	0.07
	Severe	5.38 ± 1.18 ( *n* = 134)	4.96 ± 1.31 ( *n* = 109)	4.85 ± 1.26 ( *n* = 76)	5.01 ± 1.36 ( *n* = 49)	5.33 ± 1.33 ( *n* = 26)	5.12 ± 1.7 ( *n* = 23)	0.04
	Very severe	5.64 ± 1.31 ( *n* = 90)	5.83 ± 1.3 ( *n* = 87)	5.66 ± 1.46 ( *n* = 66)	6.32 ± 1.7 ( *n* = 31)	5.36 ± 1.31 ( *n* = 17)	5.48 ± 1.93 ( *n* = 18)	0.88
	*p* -trend for age a	0.33	0.04	0.87	0.23	0.03	0.36	–
CVAI	Mild	4.46 ± 0.94 ( *n* = 20)	4.24 ± 1.15 ( *n* = 26)	4.9 ± 1.25 ( *n* = 15)	3.75 ± 0.84 ( *n* = 12)	4.64 ± 1.33 ( *n* = 6)	5.45 ± 1.66 ( *n* = 7)	0.67
	Moderate	5.16 ± 1.16 ( *n* = 102)	4.64 ± 1.27 ( *n* = 119)	4.71 ± 1.32 ( *n* = 80)	4.55 ± 1.39 ( *n* = 50)	5.01 ± 1.27 ( *n* = 29)	4.87 ± 1.3 ( *n* = 19)	0.03
	Severe	5.47 ± 1.2 ( *n* = 162)	5.4 ± 1.34 ( *n* = 127)	5.37 ± 1.42 ( *n* = 94)	5.88 ± 1.75 ( *n* = 46)	5.09 ± 1.25 ( *n* = 23)	5.6 ± 1.98 ( *n* = 23)	0.72
	Very severe	5.6 ± 1.44 ( *n* = 31)	6.3 ± 1.28 ( *n* = 17)	5.63 ± 1.23 ( *n* = 12)	6.38 ± 0.76 ( *n* = 8)	5.53 ± 1.57 ( *n* = 5)	5.23 ± 1.43 ( *n* = 5)	0.58
	*p* -trend for age a	0.06	<0.0001 b	0.003 b	0.0007 b	0.87	0.24	–
ASR	Level 1	5.24 ± 1.24 ( *n* = 176)	4.87 ± 1.37 ( *n* = 173)	5.05 ± 1.38 ( *n* = 115)	4.98 ± 1.51 ( *n* = 75)	4.85 ± 1.14 ( *n* = 37)	5.39 ± 1.81 ( *n* = 37)	0.14
	Level 2	5.25 ± 1.1 ( *n* = 94)	5.17 ± 1.39 ( *n* = 88)	5.05 ± 1.47 ( *n* = 61)	4.8 ± 1.68 ( *n* = 28)	5.3 ± 1.49 ( *n* = 21)	5.12 ± 1.39 ( *n* = 14)	0.17
	Level 3	5.78 ± 1.37 ( *n* = 39)	5.32 ± 1.09 ( *n* = 21)	5.2 ± 1.25 ( *n* = 22)	6.4 ± 2.26 ( *n* = 9)	5.71 ± 1.12 ( *n* = 4)	5.01 ± 1.37 ( *n* = 2)	0.62
	Level 4	5.67 ± 1.41 ( *n* = 6)	6.68 ± 1.49 ( *n* = 7)	6.64 ± 0.73 ( *n* = 3)	7.19 ± 0.14 ( *n* = 4)	4.07 ( *n* = 1)	4.44 ( *n* = 1)	0.5
	*p* -trend for age a	0.16	0.004 b	0.34	0.06	0.29	0.5	–
PSR	Level 1	4.75 ± 0.97 ( *n* = 14)	4.75 ± 1.25 ( *n* = 10)	4.36 ± 1.33 ( *n* = 7)	4.94 ± 2.08 ( *n* = 5)	4.69 ± 2.15 ( *n* = 3)	3.24 ± 0.07 ( *n* = 2)	0.11
	Level 2	4.8 ± 1.18 ( *n* = 50)	4.78 ± 1.42 ( *n* = 53)	4.47 ± 1.16 ( *n* = 44)	4.03 ± 1.08 ( *n* = 25)	4.93 ± 1.17 ( *n* = 12)	4.58 ± 1.35 ( *n* = 9)	0.06
	Level 3	5.08 ± 0.97 ( *n* = 94)	4.71 ± 1.36 ( *n* = 103)	4.97 ± 1.24 ( *n* = 64)	4.98 ± 1.76 ( *n* = 42)	5.13 ± 1.38 ( *n* = 20)	5.34 ± 1.26 ( *n* = 20)	0.95
	Level 4	5.68 ± 1.29 ( *n* = 157)	5.44 ± 1.32 ( *n* = 123)	5.55 ± 1.46 ( *n* = 86)	5.9 ± 1.46 ( *n* = 44)	5.08 ± 1.21 ( *n* = 28)	5.7 ± 1.96 ( *n* = 23)	0.33
	*p* -trend for age a	<0.0001 b	<0.0001 b	<0.0001 b	<0.0001 b	0.51	0.04 b	–

Abbreviations: ASR, anterior symmetry ratio; CA, cranial asymmetry; CVAI, cranial vault asymmetry index; PSR, posterior symmetry ratio.

The mean treatment duration, standard deviation, and number of patients for each subcategory are shown. The numbers of mean treatment duration are corresponding to the x-axis distance between the end points of each line in Fig. 5 . Also, the results of Kendall's rank correlation test for severity and age are shown.

a Kendall's rank correlation test.

b Indicates significance ( *p* -trend for age <0.0083 or *p* -trend for severity <0.0125).

### Safety of Helmet Therapy

Although the exact incidence is unknown because mild symptoms were often not reported by family members or not documented in the medical records, most parents reported that their infants experienced increased sweating, mild skin irritation, and rashes during helmet therapy. However, these adverse events were temporary and resolved over time, with some patients requiring ointment administration. No patients reported skin blisters or ulcers. In 13 patients (1.2%), additional helmets were required. All patients initiated helmet therapy before 6 months of age, especially eight patients were before 4 months.

## Discussion

This study confirmed significant improvements in both the 3D and 2D outcomes following helmet therapy simultaneously. In addition, the trend of younger patients or those with more severe initial conditions showing significant improvement was also validated in the 3D outcomes.

The effectiveness of helmet therapy in improving plagiocephaly observed in this study was consistent with that seen in previous studies, despite variations in study design and evaluation methods employed. 2 5 19 20 24 25 26 27 Noto et al conducted a prospective study using the same cranial orthosis (Aimet®), comparing the effectiveness of helmet therapy to a control group, and reported improvements in CA and CVAI after 2 months of treatment. 5 In our study, almost all patients underwent treatment for a duration of >2 months. We presented the final results using 3D and 2D metrics, as well as the treatment duration required for patient subgroups categorized by disease severity and age. Regarding 3D metrics, Meyer-Marcotty et al reported the effectiveness of helmet therapy using metrics similar to the anterior and posterior cranial asymmetry indices (ACAI and PCAI, respectively). 8 These indices can be converted to ASR and PSR since the alignment procedure of the 3D dataset in their study was identical to ours. Their study reported improvements in PCAI from 29.60 to 12.80 and ACAI from 3.40 to 3.00, which is equivalent to improvements in PSR from 77.16 to 88.65%, and ASR from 3.40 to 3.00%. Their study did not observe significant improvements in the frontal regions because their 20 patients had less severe frontal asymmetry. However, our study demonstrated an improvement in ASR due to the larger patient volume ( *n* = 1,038) included in our analysis.

Generally, initiating helmet therapy at an earlier age has been shown to be more effective, as demonstrated by Graham et al in their study on the correlation between age and the effectiveness of CVAI. 6 7 In our analysis, the treatment groups were divided into subgroups with narrower age and severity ranges. This analysis revealed a strong correlation between the initial age or severity and the overall effectiveness in improving both 3D symmetry and 2D metrics. These findings indicated that patients who started treatment later or had more severe conditions generally had more residual deformities after helmet therapy. This observation is important, especially for general pediatricians to consider, particularly when parents express concerns about their baby's head deformity during “infant wellness” visits. The trend of larger and faster corrections observed in younger age groups may be attributed to the growth rate of the cranial circumference during the development of a normal infant, as depicted in the normal cranial circumference growth chart commonly published by the Japanese Ministry of Health, Labor, and Welfare. It is important to avoid missing valuable opportunities for treatment, as they may become irrecoverable. Therefore, clinical attention and intervention may be necessary to address severe cranial deformities at a younger age or before they worsen.

While Graham et al found that patients with more severe deformities generally require longer treatment durations, we observed a different trend in our patients. 7 This discrepancy can be attributed to our practice of presenting families with the results of a 3D scan. This immediate visualization of objective improvements in the head shape of their infants may lead families of older infants to be satisfied with the treatment and choose to discontinue it, particularly if they see objective improvements in the shape of their infant's head, or if there is no significant clinical growth. However, for younger infants, many parents may still have concerns about the potential relapse of head flattening, and may prefer to continue with the treatment.

As demonstrated by Kato et al, there are cases where patients have normal or mild CA or CVAI but severe ASR or PSR. 10 From our perspective, it is still worth treating these patients due to the asymmetry that concerns the parents, even though the natural course of such cases remains unknown. While the use of 3D scanning for assessing the asymmetry of the head shape from multiple angles is not yet widespread in Japan, it offers the advantage of objective comparisons between different time points. Therefore, the ideal approach is to use both 2D and 3D evaluations to accurately assess the severity and improvement of deformities.

Our results help provide valuable guidance for clinicians initiating helmet therapy and inform parents about the expected treatment effectiveness. Fig. 5 shows the mean improvement in each outcome based on the age of the patients and the severity of the condition. Additionally, the right panel indicates that delaying treatment until an older age results in a loss of potential improvement. By having realistic expectations, parents and clinicians can make informed decisions regarding the pursuit of treatment. It is important to note that postscan analysis is required to obtain accurate measurements of ASR and PSR. It may not be possible to use it for immediate same-day clinical evaluation unless the facility has specialized staff, such as at our clinic.

Similar to most other studies, there were a few critical aspects that could not be eliminated. First, it should be noted that this was a single-arm, nonrandomized study without a control group of untreated patients. Therefore, further studies are required to assess the natural course of cranial deformation in untreated infants. Second, we did not assess the duration of daily helmet use. Although parents were instructed to have their child wear a helmet for 23 hours a day, the actual duration varied among the patients, which could have potentially affected the effectiveness of the treatment. Objective recording of wearing time is challenging, and further technological advancements are necessary to accurately collect data on duration of use and improve compliance. Lastly, the results were not followed-up on in the long term. Therefore, it is impossible to determine the duration of correction in relation to the age and severity at which helmet therapy was initiated.

In conclusion, our study highlighted significant differences between age groups within each severity group as well as between severity groups within each age group. These findings emphasize the importance of both age and disease severity as crucial factors in determining the outcomes of helmet therapy. Therefore, we would like to propose the following wording as a new guideline: “Since the amount of improvement with helmet therapy depends on the age before treatment, children with deformities should be promptly informed of the existence of helmet therapy options. Since the amount of improvement by helmet therapy depends on the age and severity before treatment, it is necessary to consult a specialist with proven experience and be informed of the expected degree of improvement for individual patients.”
